# The Replicase Gene of Avian Coronavirus Infectious Bronchitis Virus Is a Determinant of Pathogenicity

**DOI:** 10.1371/journal.pone.0007384

**Published:** 2009-10-09

**Authors:** Maria Armesto, Dave Cavanagh, Paul Britton

**Affiliations:** Division of Microbiology, Institute for Animal Health, Compton Laboratory, Compton, Newbury, Berkshire, United Kingdom; Institut Pasteur Korea, Republic of Korea

## Abstract

We have previously demonstrated that the replacement of the S gene from an avirulent strain (Beaudette) of infectious bronchitis virus (IBV) with an S gene from a virulent strain (M41) resulted in a recombinant virus (BeauR-M41(S)) with the *in vitro* cell tropism of the virulent virus but that was still avirulent. In order to investigate whether any of the other structural or accessory genes played a role in pathogenicity we have now replaced these from the Beaudette strain with those from M41. The recombinant IBV was in effect a chimaeric virus with the replicase gene derived from Beaudette and the rest of the genome from M41. This demonstrated that it is possible to exchange a large region of the IBV genome, approximately 8.4 kb, using our transient dominant selection method. Recovery of a viable recombinant IBV also demonstrated that it is possible to interchange a complete replicase gene as we had in effect replaced the M41 replicase gene with the Beaudette derived gene. Analysis of the chimaeric virus showed that it was avirulent indicating that none of the structural or accessory genes derived from a virulent isolate of IBV were able to restore virulence and that therefore, the loss of virulence associated with the Beaudette strain resides in the replicase gene.

## Introduction

Avian infectious bronchitis virus (IBV) is a member of the genus *Coronavirus*, family *Coronaviridae*, order *Nidovirales* [1], [2]. Together with the genetically closely related Turkey coronavirus [3]–[6], Pheasant coronavirus [7], and recently identified coronaviruses from several species of wild birds [8], [9], a beluga whale [10] and an Asian Leopard cat [11] form the Group 3 coronaviruses. IBV is a highly infectious pathogen of domestic fowl that replicates primarily in the respiratory tract but also in epithelial cells of other organs, including the gut, kidney and oviduct [12]–[14], and is the causative agent of infectious bronchitis, a disease that is responsible for economic losses in the poultry industry throughout the world [15].

Coronaviruses are enveloped viruses that replicate in the cell cytoplasm and contain a single-stranded, positive-sense RNA genome of 28 to 32 kb [16]. IBV has a 27.6 kb RNA genome and like all coronaviruses contains the four structural proteins; spike glycoprotein (S), small membrane protein (E), integral membrane protein (M) and nucleocapsid protein (N) which interacts with the genomic RNA. All coronaviruses also encode a set of accessory protein genes of unknown function that are not required for replication *in vitro* [17]–[23], but may play a role in pathogenesis [19], [22]. IBV encodes two accessory genes, genes 3 and 5, which both express two accessory proteins 3a, 3b and 5a, 5b, respectively. In addition to the structural and accessory genes, two-thirds of a coronavirus genome comprises the replicase gene, which expresses two polyproteins, pp1a and pp1ab, in which pp1ab is an extension product of pp1a as a result of a -1 ribosomal shift mechanism. The two polyproteins are cleaved by two types of virus-encoded proteinases usually resulting in 16 non-structural proteins (Nsp1-16); IBV lacks Nsp1 thereby encoding Nsp2-16.

We have previously shown that the IBV accessory genes are not required for replication *in vitro* [17], [18]; however, we could not determine any role of the IBV accessory proteins in pathogenicity as our reverse genetics system is based on the avirulent Beaudette strain. Replacement of the Beaudette S gene with the corresponding M41 S gene sequence altered the tropism of the rIBV but did not result in a change in virulence [24]–[26]. This implied that although the IBV S gene may play a role in virulence, associated with tropism, expression of an S gene from a virulent strain alone was not sufficient to alter the avirulent phenotype associated with Beaudette.

Infectious bronchitis is mainly controlled by the use of live attenuated vaccines derived from virulent viruses by multiple serial passages, usually greater than 50 passages, in 10-11-day-old embryonated chicken eggs [13], [27]–[29]. As a consequence of this process the virus becomes more adapted for the embryo, reflected by more efficient replication and higher pathogenicity for the embryo, with concomitant attenuation for chickens and in some cases loss of immunogenicity. However, the mutations associated with attenuation of pathogenicity for the chicken are unknown and variable leading to differing efficacies associated with different vaccines.

Our previous studies have shown that replacement of the S gene from the avirulent Beaudette isolate with that from a virulent virus (M41) did not restore virulence but did alter the tropism of the rIBV and restore immunogenicity for subsequent challenge with M41. Previously Beaudette had been considered to be poorly immunogenic and never used as a vaccine strain [30]. In this study, we describe the generation of recombinant IBVs that consisted of the replicase gene from the avirulent Beaudette strain and the structural and accessory genes from the virulent M41 isolate of IBV, to determine whether the replicase or the combination of the structural and accessory genes of IBV play a role in pathogenesis.

## Materials and Methods

### Cells and viruses

The growth of IBV in chick kidney (CK) cells was as described previously [31]–[33]. The IBV isolates used were: (1) Beaudette-CK (Beau-CK; [34]), a virus adapted for growth in CK cells that can grow on but has not been adapted for growth in Vero cells, an African green monkey cell line; (2) Beau-R, a recombinant IBV (rIBV) produced from a full-length cDNA of Beau-CK using our IBV reverse genetics system [35]; and (3) M41-CK, an isolate derived from M41 [36] following adaption to growth on CK cells. Both the Beaudette and M41 strains of IBV belong to the same, Massachusetts, serotype. All IBV strains were titrated in CK cells. Vaccinia viruses were grown and titrated on Vero cells and large stocks for DNA isolation were grown in BHK-21 cells [37]. All nucleotide and amino acid residue numbers refer to the positions in IBV Beau-R [35] accession N^o^ AJ311317.

### Construction of modified IBV cDNA

**Figure 1:**
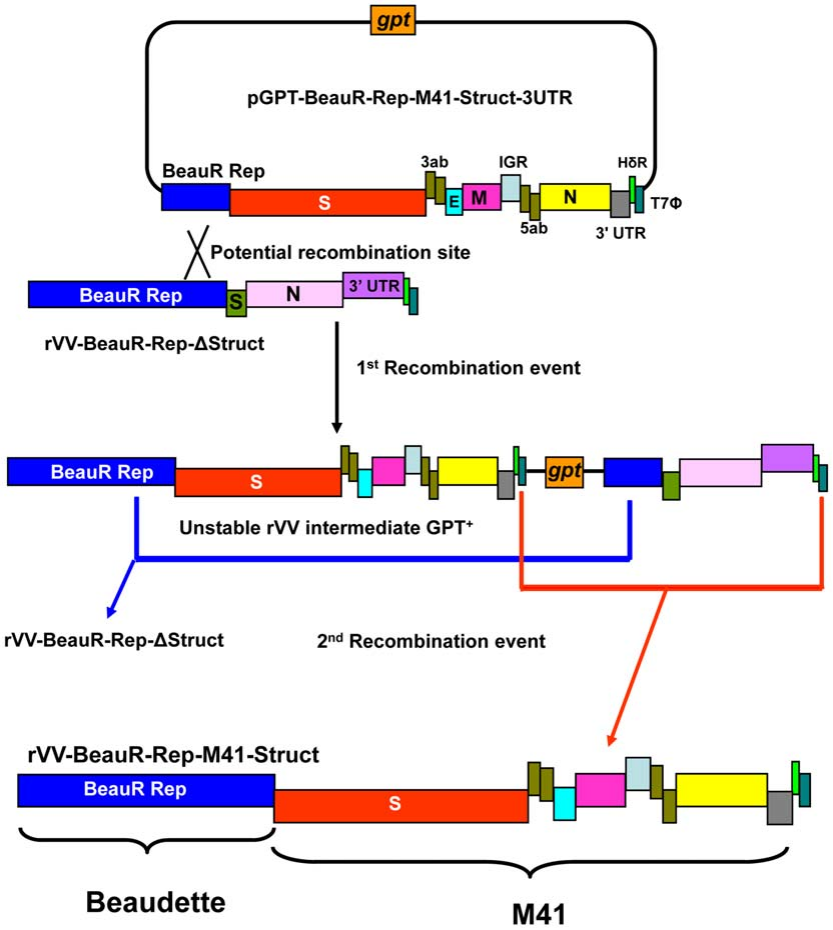
The transient dominant selection process for modifying the truncated Beau-R cDNA within vaccinia virus rVV-BeauR-Rep-Δ-Struct. The M41-CK-derived cDNA, representing the M41 structural and accessory genes and the M41 3′-UTR, within pGPT-BeauR-Rep-M41-Struct-3UTR was fused to the Beau-R replicase gene in the rVV by a homologous recombination event between the Beau-R replicase sequence common to both constructs. A potential rVV, rVV-BeauR-Rep-M41-Struct, containing a full-length IBV cDNA with the replicase gene from Beau-R and the rest of the genome from M41-CK was isolated following the TDS process. The complete plasmid DNA was fused to the truncated Beau-R cDNA by a single-step homologous recombination event; via the Beau-R replicase sequence common to both sequences. The initial resultant rVVs had a GPT^+^ phenotype allowing selection in the presence of mycophenolic acid. Removal of mycophenolic acid resulted in two types of spontaneous intramolecular recombination events, due to the instability of the IBV cDNA with tandem repeats of similar sequences, resulting in either rVV-BeauR-Rep-Δ-Struct (no modification) or in rVV-BeauR-Rep-M41-Struct; the desired rVV. Both recombination events resulted in the loss of the GPT gene. The IBV genes representing the structural and accessory genes are shown along with the 3′-UTRs and IGR sequences, a potential recombination event is indicated between the 1416 nt of the Beau-R replicase gene sequence common to both constructs.

The region of the IBV M41 genome corresponding to the structural, accessory genes and the 3′-UTR was ligated onto the last 1416 nt of the Beau-R replicase gene and inserted into *Hin*dIII and *Sal*I digested pGPT-NEB193 [25]. The resulting plasmid, pGPT-BeauR-Rep-M41-Struct-3UTR, consisted of the 3′-end of the replicase gene of Beau-R, and the region of the M41-CK genome encoding the structural and accessory genes terminated by the M41-CK derived 3′UTR (Fig. 1).

### Generation of recombinant vaccinia viruses containing modified IBV cDNAs

The IBV cDNA within pGPT-BeauR-Rep-M41-Struct-3UTR was introduced, by homologous recombination using the transient dominant selection (TDS) ([25], [37]), into the IBV Beaudette cDNA within the vaccinia virus genome in rVV-BeauR-Rep-ΔStruct containing Beau-R-derived sequence corresponding to the replicase gene followed by the first 376 nt of the S gene, part of the N gene and the 3′-UTR (Fig. 1). Briefly, 50% confluent monolayers of vero cells were infected with rVV-BeauR-Rep-ΔStruct, containing the Beau-R cDNA sequence, at a MOI of 0.2 and transfected 2 h later with 5 µg of pGPT-BeauR-Rep-M41-Struct-3UTR in lipofectin (Invitrogen). Resultant phenotypically GPT^+^ rVVs were selected by three rounds of plaque purification using vero cells in the presence of 25 µg/ml mycophenolic acid (MPA), 250 µg/ml xanthine and 15 µg/ml hypoxanthine. Randomly selected MPA resistant rVVs were grown and plaque purified three times using vero cells in the absence of selection medium. This resulted in a second recombination event (see Fig. 1) involving the loss of the GPT gene from the rVVs, and either generation of an IBV cDNA corresponding to the sequence in rVV-BeauR-Rep-ΔStruct or a full-length IBV cDNA consisting of a Beau-R replicase gene and the rest of the IBV genome derived from IBV M41-CK within the VV genome (Fig. 1). PCR was used to confirm the absence of the GPT gene in resulting rVVs, which were further screened by PCR amplification of the IBV 3′-UTR, using oligonucleotides BG56 (5′-^26941^CAACAGCGCCCAAAGAAG^26958^ ) and 93/100 (5′-^27607^GCTCTAACTCTATACTAGCCT^27587^-3′), part of the replicase gene, using oligonucleotides BG40 (5′-^18941^ATCTAATTTGCTCGTTCA^18958^-3′ and BG128 (5′-^19720^CGCCACTCCTTTGTCGCTTC^19739^-3′, and the junction of the replicase and S genes using oligonucleotides BG40 (5′-^18941^ATCTAATTTGCTCGTTCA^18958^-3′ and BG134 (5′-^21398^AGCAATTGAAACTGAAAGTG^21417^-3′. Oligonucleotides BG-56 and 93/100 were used to discriminate between the 3′-UTRs from Beau-R and M41 derived sequences; the 3′-UTR from Beau-R results in a 667 bp product but a 483 bp PCR from M41-CK, due to a 184 nt deletion in the M41 3′-UTR. A rVV, rVV-BeauR-Rep-M41-Struct, that contained a full-length IBV cDNA consisting of the replicase gene from Beau-R and the rest of the genome derived from IBV M41-CK was identified and used for further work.

### Recovery of infectious IBV

Recombinant vaccinia virus DNA from rVV-BeauR-Rep-M41-Struct containing the BeauR-Rep-M41-Struct chimaeric full-length IBV cDNA was purified and used for the rescue of rIBVs in CK cells using rFPV/T7 [38] for the generation of infectious IBV RNA ([25], [35], [37]). Resultant chimaeric rIBVs were passed three times in CK cells before being used in subsequent experiments. Total cellular RNA was extracted from IBV-infected CK cells using the RNeasy method (Qiagen) and RT-PCR (Ready-To-Go^TM^ RT-PCR beads) for amplification of the 3′-UTR of the rIBV-derived RNA, using oligonucleotides BG 56 and 93/100, to confirm the identity of the rIBV.

### Multiple-step growth curves of rIBVs in CK cells

Confluent monolayers of CK cells in 6-well plates were infected with viruses at a MOI of 0.1 PFU in triplicate for each time point. Following attachment, for 1 h at 37°C, the cells were washed twice with phosphate-buffered saline (PBS) to remove residual virus and incubated at 37°C. Samples of media were collected at 24, 48, 72 and 96 h post-infection and assayed in triplicate for progeny virus by plaque assay using CK cells.

### Growth kinetics of rIBVs in tracheal organ cultures

Chicken tracheal organ cultures (TOCs) were prepared from 19-day-old specific pathogen free (SPF) Rhode Island Red chicken embryos [39]. Groups of five TOCs, in triplicate, were inoculated with 0.5 ml of medium containing 5.4×10^4^ PFU/ml of each virus. After incubation at 37°C for 1 h, the inoculum was removed and the TOCs were washed three times with PBS, 1 ml of medium was added and the TOCs were incubated at 37°C until the samples were taken. At the selected time points medium from the TOCs was removed and analysed for progeny virus by plaque titration on CK cells.

### *In vivo* analysis of the rIBVs

All experiments were carried out in accordance with the UK Home Office guidelines using SPF Rhode Island Red chickens obtained from the Poultry Production Unit of the Institute for Animal health. Four groups (n = 12) of 1-day-old SPF Rhode Island Red chickens were inoculated via the conjunctival (eye drop) and intranasal routes with 10^6^ PFU/ml of each virus in a total of 0.1 ml serum-free BES (N,N-Bis(2-hydroxyethyl)-2-aminoethanesulphonic acid) medium. The chickens were housed in positive-pressure, HEPA-filtered isolation rooms, and each group was housed in a separate room. The birds in the mock-infected group were inoculated with serum-free BES medium.

### Assessment of pathogenicity

The IBV-associated clinical signs used to determine pathogenicity were snicking, tracheal rales (a sound emanating from the bronchi, also detected by vibrations when holding a chick), wheezing (dyspoena), nasal discharge, watery eyes and ciliary activity of the trachea [26]. Chicks were observed daily for clinical signs; snicks (a sound similar to a sneeze) were counted by two persons over 2 minutes. Birds were checked individually for the presence of tracheal rales, nasal discharge, watery eyes and wheezing. Tracheas were removed from three randomly selected chickens from each group at 4 and 7 days post-inoculation for assessment of ciliary activity. Ten 1 mm sections were cut from three different regions of each trachea and the level of ciliostasis of each tracheal section was determined.

### Virus replication in chickens

The remaining regions of the tracheas from the infected birds were cut longitudinally and the epithelial cells scraped from the tracheas and transferred to 1 ml PBS. The samples were analysed for the presence of viable IBV by titration in TOCs or used for RNA extraction using the RNeasy method and analysed by RT-PCR using oligonucleotides BG56 and 93/100 to determine the identity of the 3′-UTR.

### Serial passage of rBeauR-Rep-M41-Struct

Confluent monolayers of CK cells were used for serial passage of rBeauR-Rep-M41-Struct 25 times. Briefly, cells were infected with the rIBV and 24 h post-infection medium was collected, diluted 1∶10 and used to infect a new monolayer of CK cells. This process was repeated until passage 25 (P_25_). Total RNA was extracted from the P_25_ infected CK cells and RT-PCR was used to generate a series of overlapping PCR products covering the complete genome of the P_25_ rIBV. The RT-PCR products were sequenced using a variety of oligonucleotides, derived from the Beau-CK sequence [40]. Assembly of the sequences was performed using Gap4 of the Staden Sequence Software Programs [41].

## Results

### Generation of chimaeric rIBVs with the replicase gene from Beaudette and the rest of the genome M41-CK

A full-length IBV-derived cDNA, within the vaccinia virus genome, was generated by homologous recombination using the TDS method and consisted of the replicase gene from the apathogenic IBV strain Beau-R and the structural and accessory genes plus the 3′-UTR from the pathogenic M41 strain of IBV. This was achieved using a Beau-R-based receiver sequence consisting of the complete replicase gene, followed by the first 376 nt of the S gene fused to the N gene and 3′-UTR (Fig. 1). The donor sequence consisted of the last 1246 nt of the Beau-R replicase gene fused to M41-CK-derived cDNA from the S gene to the poly(A) tail (Fig. 1).

Following TDS, DNA was extracted from 20 rVVs, potentially containing the chimaeric IBV full-length cDNA. Analysis by PCR, using GPT specific primers to confirm the loss of the *E. coli* GPT gene, on six of the rVV DNAs confirmed the loss of the GPT gene following the second TDS recombination event (Fig. 1). The IBV cDNAs within these six rVV DNAs were analysed (1) for the presence of the M41-CK-derived 3′-UTR sequence, which is 184 nt shorter than the Beau-R 3′-UTR [42], (2) the junction between the replicase and S gene using oligonucleotides BG40 and BG134 and (3) for the presence of the M41-CK-derived M gene using oligonucleotides BG52 (5′-^24945^GAATGGTGTTCTTTATTG^24962^-3′) and BG146 (5′-^25549^ TCTAACACTCTAAGTTGAG^25567^-3′); to confirm that the second TDS recombination event had not resulted in generation of the starting receiver sequence (Fig. 1). Two rVVs, rVV-BeauR-Rep-M41-Struct-2 and rVV-BeauR-Rep-M41-Struct-12, that did contain the M41-CK-derived 3′-UTR and M gene were further screened by spot sequence analysis of the IBV cDNA to confirm that the region downstream of the Beau-R replicase gene had been replaced with the corresponding sequence from M41-CK; the sequences were as expected for the required chimaeric IBV sequence.

### Recovery of infectious rIBVs from the rVVs

Two infectious rIBVs, rBeauR-Rep-M41-Struct-2 and rBeauR-Rep-M41-Struct-12, were recovered from DNA extracted from rVV-BeauR-Rep-M41-Struct-2 and rVV-BeauR-Rep-M41-Struct-12, respectively, using CK cells, previously infected with rFPV/T7, to provide T7 RNA polymerase, and co-transfected with the rVV DNA and pCi-Nuc [35]. The transfected CK cells (P_0_) were incubated until they showed a cytopathic effect (CPE), the medium was filtered to remove any rFPV/T7 and any potential rIBV passaged three times more on CK cells (P_3_). Total RNA from the infected P_3_ CK cells was extracted and analysed by RT-PCR and spot sequence analysis. Sequence analysis showed that rBeauR-Rep-M41-Struct-12 contained an extra adenosine nucleotide at position 25317 in a six base polyadenosine repeat sequence within the M41 intergenic region (IGR) between the end of the M gene and start of gene 5. Consequently only rBeauR-Rep-M41-Struct-2 from the P_3_ CK cells was titrated in CK cells and used for further characterisation. Sequence analysis of the M41-CK-derived sequence in rBeauR-Rep-M41-Struct-2 revealed three nucleotide changes when compared to the sequence in rVV-BeauR-Rep-M41-Struct-2 (Table 1). The nucleotide changes resulted in one amino acid change in the S protein, the other was a silent mutation and a single amino acid change in the N gene. Comparison of the sequence indicated that the changes arose during rescue or during the first three passages of the rescued viruses.

**Table 1:** Nucleotide substitutions identified in the rIBVs when compared to the IBV sequence in rVV-BeauR-Rep-M41-Struct-2.

IBV gene	Genome position^a^	Nucleotide change	Codon change	Amino acid substitution	Position in protein	Occurrence of substitution
S	20467	U→C	UUU→CUU	Phe→Leu	34	P_3_→P_25_
	20676	C→U	AAC→AAU	Silent Asn	103	On rescue
	22266	C→A	GAC→GAA	Asp→Glu	633	On rescue
5b	25815	U→A	UUU→UAA	Phe→Stop	30	P_3_→P_25_
	25816	U→A				
N	26172	C→A	AAC→AAA	Asn→Lys	85	On rescue

a based on the sequence of IBV Beau-R accession No AJ311317.

### Characterisation of the rIBV *in vitro*

**Figure 2:**
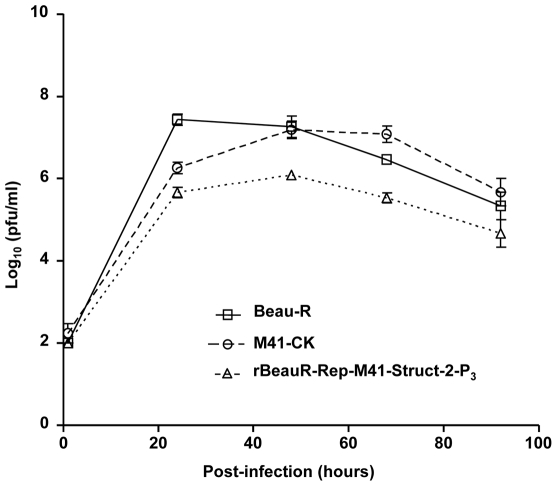
Growth profile of rIBV rBeauR-Rep-M41-Struct-2-P_3_ on CKCs. Monolayers of CKCs were infected with Beau-R, M41-CK or rIBV rBeauR-Rep-M41-Struct-2-P_3_ at a MOI of 0.1 PFU/cell. After an adsorption period of 1 h at 37°C, the cells were washed and incubated for 96 h. At selected time points post-infection cell medium was analysed for progeny virus by plaque titration assay on CKCs. Each titration value is the mean of three replicates and standard error values are shown as bars.

The growth characteristics of rBeauR-Rep-M41-Struct-2 were investigated and compared to Beau-R and M41-CK *in vitro* using CK cells infected with 2×10^5^ PFU (MOI ≈0.1). The titres of the progeny viruses were analysed over a 96 h post-infection period. As can be seen from Fig. 2, Beau-R reached peak titres by 24 h post-infection whereas M41-CK and rBeauR-Rep-M41-Struct-2 reached peak titres by 48 h post-infection. However, whereas M41-CK reached a similar titre as Beau-R by 48 h post-infection, the titre of rBeauR-Rep-M41-Struct-2 was approximately 1 log_10_ lower and remained lower over the rest of the time course.

### Characterisation of the rIBV *in vivo*

**Figure 3:**
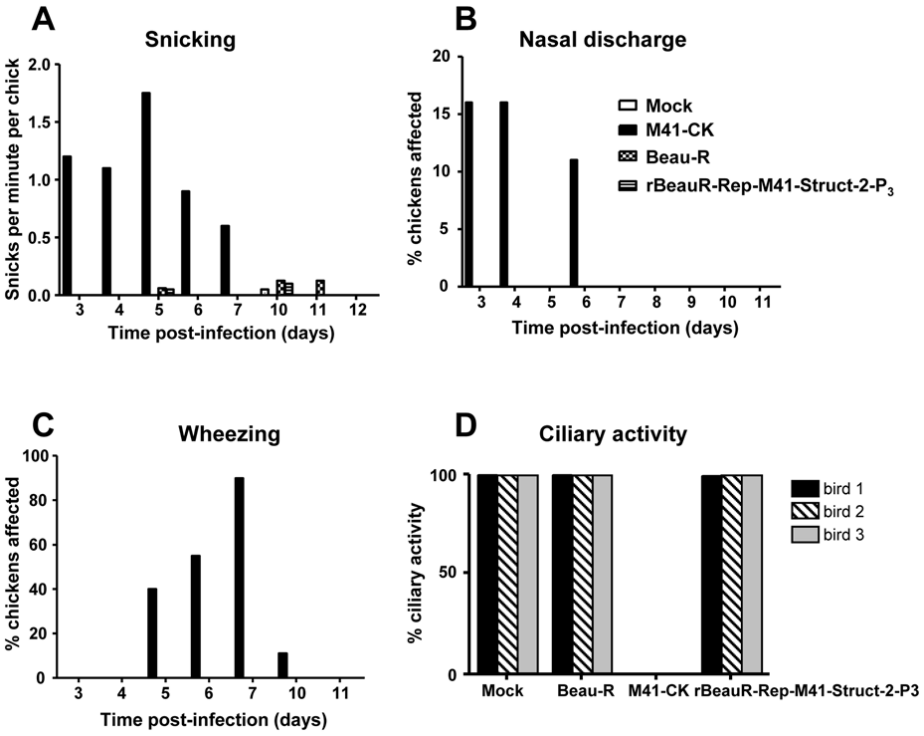
Comparison of the pathogenicities of Beau-R, M41-CK and rBeauR-Rep-M41-Struct-2-P_3_. Four groups (n = 12) of 1-day-old SPF chicks were inoculated intranasally/eye drop with 0.1 ml of 10^6^ PFU/ml of each virus. Mock-infected chicks were inoculated with 0.1 ml of medium. Clinical signs were recorded from days 3 to 11 post-infection in which clinical signs were recorded for individual birds, apart for snicking where signs were observed from a group. The clinical signs recorded were (A) snicking, (B) nasal discharge (mean of all birds), (C) wheezing (mean of all birds) and (D) ciliary activity in tracheal rings (mean of 10 tracheal rings from three individual chicks per group).

Twelve one-day-old SPF chickens, in four groups, were inoculated with 0.1 ml of 10^6^ PFU/ml of rBeauR-Rep-M41-Struct-2, M41-CK or Beau-R or using 0.1 ml serum-free medium (mock infection) by eye-drop and intranasally. The birds were observed for clinical signs up to 11 days post-inoculation. At 4 and 7 days post-infection the tracheas of three randomly selected chickens from each group were examined for ciliary activity and presence of IBV. Observations for clinical signs, snicking, wheezing and nasal discharge, of an IBV infection were carried out daily on each group from three days post-infection. Only chickens inoculated with M41-CK showed any clinical signs of an IBV infection. As can be seen from figure 3, M41-CK induced high rates of snicking, which peaked by day 5 post-inoculation, whereas Beau-R and rBeauR-Rep-M41-Struct-2 induced a much lower rate of snicking, similar to those observed for the mock-inoculated chickens (Fig. 3A). Similarly, only chickens (11–16%) inoculated with M41-CK showed any nasal discharge three, four and six days post-inoculation (Fig. 3B). Again, wheezing was only observed in chickens inoculated with M41-CK, in which 90% of the infected chickens demonstrated wheezing by 7 days post-inoculation (Fig. 3C). Analysis of the tracheas isolated from the chickens showed that the mock, Beau-R and rBeauR-Rep-M41-Struct-2 infected chickens had >95% ciliary activity whereas the chickens inoculated with M41-CK showed 0% ciliary activity (100% ciliostasis; Fig. 3D). In summary, our observations of the parameters used to assess pathogenicity demonstrated that rBeauR-Rep-M41-Struct-2 was not pathogenic and that it had the characteristics associated with Beau-R rather than M41-CK.

### Presence of IBV in the tracheas from the infected chickens

In order to determine whether there was any virus in the tracheas of the infected chickens, the epithelial cells were scraped from the tracheas taken from three chickens from each group four days post-infection and any virus present titrated in TOCs. The average titre of M41-CK, isolated from the tracheas of the three chickens, was 4.1 log_10_ CD_50_/ml, in contrast no detectable virus was isolated from the tracheas taken from the chickens infected with either Beau-R or rBeauR-Rep-M41-Struct-2. This result indicated that if any virus was present in the tracheas of the chickens infected with Beau-R or rBeauR-Rep-M41-Struct-2 the amounts present were at least 4 log_10_ CD_50_/ml lower than the M41-CK detected from the tracheas of the chickens infected with M41-CK.

### Analysis of IBV-RNA present in the tracheas of infected chickens

**Figure 4:**
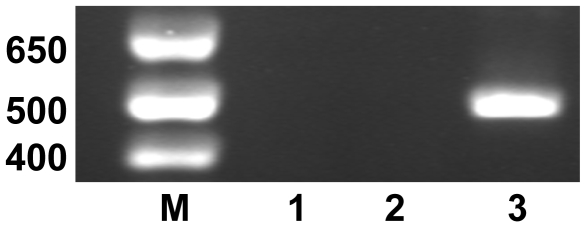
Detection of IBV-derived RNA by RT-PCR in tracheal epithelial cells of infected chicks. Tracheas were removed 4 days post-infection and epithelial cells were scraped from sections of the tracheas and used for total RNA extraction using RNeasy kit (Qiagen). The RT-PCR analyses were performed using oligonucleotides located at the end of the N gene and at the end of the 3′-UTR and analysed on 1% agarose gels in which lane (M) contained 1 Kb Plus DNA Ladder marker; and lanes 1–3 corresponded to tracheal epithelial cell extracts from chicks infected with (1) Beau-R; (2) rBeauR-Rep-M41-Struct-2-P_3_ and (3) M41-CK. An RT-PCR product of 483 nt was indicative of M41-derived RNA (M41-CK and rBeauR-Rep-M41-Struct-2-P_3_) and a product of 667 nt was indicative of Beau-R.

Analysis of the tracheal epithelial cells isolated from the infected chickens, for the presence of IBV by titration on TOCs, had indicated that either there was no Beau-R or rBeauR-Rep-M41-Struct-2 present or that the levels of both viruses were below detection. Our previous results had shown that Beau-R was not reliably detected in the tracheas of Beau-R in infected chickens whereas M41-CK is detectable [26], [43]. To check for the presence of M41-CK-, Beau-R- or rBeauR-Rep-M41-Struct-2-derived RNA in the tracheal epithelial cells from the infected chickens, total RNA was isolated from the epithelial cells and analysed by RT-PCR using oligonucleotides BG56 and 93/100, corresponding to the 3′-UTR of the IBV genomes. As can be seen from Fig. 4, the tracheal epithelial cells isolated from chickens infected with M41-CK were positive for the presence of M41-CK-derived RNA. In contrast, no IBV-derived RNA was detected in the tracheal epithelial cells of chickens infected with either Beau-R or rBeauR-Rep-M41-Struct-2 supporting our virus isolation data. Furthermore, sequence analysis of the IBV-derived RT-PCR product amplified from the tracheal epithelial cells of chickens infected with M41-CK confirmed that it was derived from IBV M41-CK. This was consistent with previous results suggesting that Beau-R probably does not reach the tracheas of chickens infected by the eye-drop and intranasal routes and that similarly rBeauR-Rep-M41-Struct-2 did not appear to reach the epithelial cells of the tracheas of infected chickens. A rIBV, BeauR-M41(S) [24], which consisted of the Beau-R genome but with an M41-derived S gene, had the tropism of M41 *in vitro* but the characteristics of Beau-R *in vivo* indicating, together with our rBeauR-Rep-M41-Struct-2 result, that the inability of Beau-R-derived viruses to reach the tracheas of infected chickens resides within the replicase gene.

### Characterisation of the rIBV *ex vivo*

**Figure 5:**
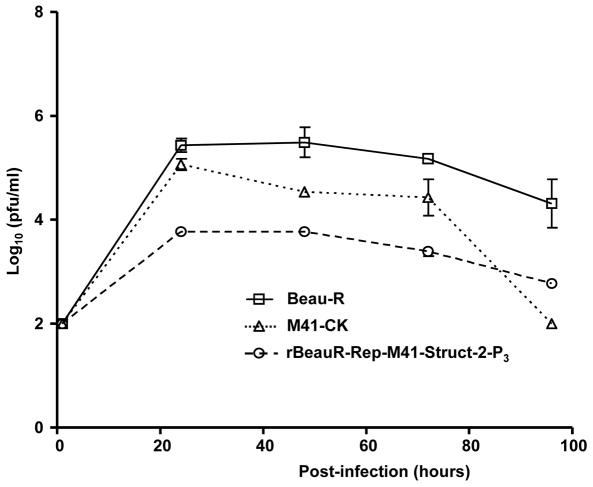
Growth profile of rIBV rBeauR-Rep-M41-Struct-2-P_3_ in TOCs. Groups of five TOCs were infected in triplicate with 0.5 ml of 5.4×10^4^ PFU/ml of Beau-R, M41-CK or rIBV rBeauR-Rep-M41-Struct-2-P_3_. After 1 h at 37°C, the medium was removed and the TOCs were washed 3 times with PBS and incubated in 1 ml of medium for 96 hours. At selected time points post-infection medium was analysed for progeny virus by plaque titration assay on CKCs. Each titration value is the mean of three replicates and standard error values are shown as bars.

Infection of tracheal epithelial cells *ex vivo* is a method of growing IBV strains that have not been adapted for growth either in embryonated eggs or primary cell cultures and results in cessation of ciliary activity (ciliostasis) of the epithelial cells. Previous work demonstrated that our rIBVs, Beau-R and BeauR-M41(S), caused ciliostasis of infected TOCs with concomitant production of progeny virus [26]. Analysis of tracheal epithelial cells from chickens infected with rIBV-BeauR-Rep-M41-Struct-2 had shown that there was no virus present. Therefore, we decided to investigate the replication of rBeauR-Rep-M41-Struct-2, for comparison with Beau-R and M41-CK, *ex vivo* in TOCs. Progeny viruses in TOC medium taken from each group of infected TOCs at the specific time points were titrated by plaque assay on CK cells to investigate the growth kinetics of the three IBVs in TOCs. As can be seen from Fig. 5 all three viruses replicated in the TOCs. Although the viruses reached maximum titres by 24 h post-infection, the titre of rBeauR-Rep-M41-Struct-2 was between 1-2 log_10_ units lower than the titres observed for M41-CK and Beau-R, respectively, at this and most later time points. Interestingly, although rBeauR-Rep-M41-Struct-2 replicated and produced progeny virus in TOCs there was no observable ciliostasis when compared to the growth of Beau-R and M41-CK.

### Serial passage of rBeauR-Rep-M41-Struct

Following rescue of rBeauR-Rep-M41-Struct-2 all growth experiments were carried out using virus that had been passaged three times (P_3_) in CK cells. We routinely use the P_3_-derived rIBVs so that there is a lower probability that changes will have occurred within the virus genome, as with other positive strand RNA viruses multiple passage will result in changes in both nucleotides and amino acids; for example the acquisition of the extra adenosine residue in rBeauR-Rep-M41-Struct-12. The rIBV, rBeauR-Rep-M41-Struct-2, showed slightly reduced growth properties when compared with both parental viruses, Beau-R and M41-CK. Surprisingly, although rBeauR-Rep-M41-Struct-2 grew in TOCs it did not cause ciliostasis, which is induced by both parental viruses. Due to the chimaeric nature of the rIBV the 5′-UTR, including the leader sequence, corresponds to Beau-R, with the 3′-UTR derived from M41-CK. There are known nucleotide differences in these regions of the IBV genome between the two viruses ([33], [42]. This introduces the possibility that if the two UTRs interact this could affect the growth properties of the recombinant viruses. As there are only 7 and 3 nucleotide difference between the 5′-UTR and the conserved region of the 3′-UTR, respectively, from Beau-R and M41-CK we decided to serially passage rBeauR-Rep-M41-Struct-2 25 times on CK cells to see whether this would result in a virus with a higher growth rate and that could cause ciliostasis in TOCs and to determine in the P_25_-derived virus whether this was a result of any nucleotide substitutions in either of the UTRs or any other region of the genome that may account for any potential change in phenotype.

**Figure 6:**
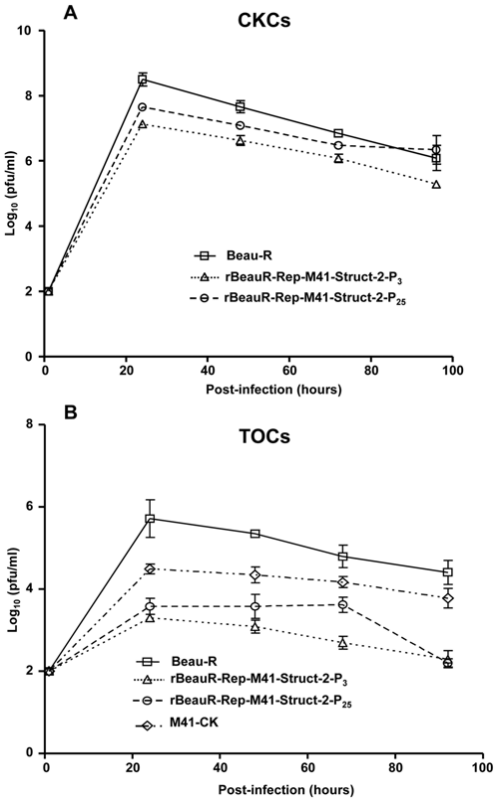
Growth profiles of the chimaeric rIBVs rBeauR-Rep-M41-Struct-2-P_3_ and rBeauR-Rep-M41-Struct-2-P_25_ in CKCs and TOCs. Following serial passage of rBeauR-Rep-M41-Struct-2-P_3_ in CKCs the growth profiles of virus rBeauR-Rep-M41-Struct-2-P_25_ from passage 25 were compared to those of Beau-R and rBeauR-Rep-M41-Struct-2-P_3_ in (A) CKCs and (B) TOCs to determine whether passage resulted in an altered growth profile. The viruses were analysed as described in Fig. 2 for CKCs and in Fig. 5 for TOCs.

The growth characteristics of rBeauR-Rep-M41-Struct-2-P_25_ were initially examined in CK cells and compared to rBeauR-Rep-M41-Struct-2-P_3_. As can be seen from Fig. 6A the peak titre of rBeauR-Rep-M41-Struct-2 -P_25_ was higher, approximately 0.5 log_10_ unit, than for rBeauR-Rep-M41-Struct-2 -P_3_ at 24 h post-infection and remained higher throughout the time course. Subsequently, the growth properties of rBeauR-Rep-M41-Struct-2-P_25_ were then studied in TOCs and as can be seen from Fig. 6B rBeauR-Rep-M41-Struct-2-P_25_ rIBV grew to a higher titre than rBeauR-Rep-M41-Struct-2-P_3_, reaching a 1 log_10_ unit difference by 72 h post-infection, although the two viruses had a similar titre by 96 h post-infection. However, analysis of TOCs infected with rBeauR-Rep-M41-Struct-2-P_25_ also showed that this virus did not cause ciliostasis.

**Figure 7:**
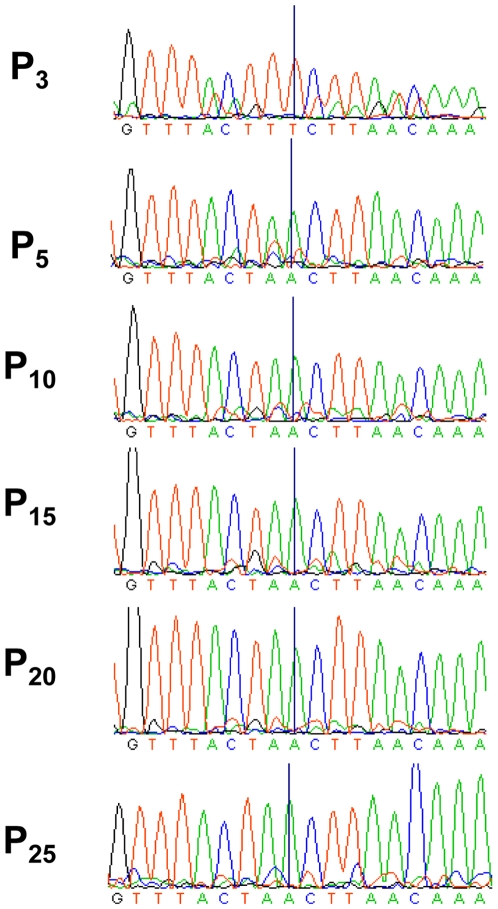
Acquisition of the premature stop codon identified in ORF 5b following serial passage of rBeauR-Rep-M41-Struct-2-P_3_. Sequence analysis of rBeauR-Rep-M41-Struct-2-P_25_ identified a di-nucleotide substitution, UU→AA, which resulted in a premature stop codon in ORF 5b. The figure compares the sequence chromatograms showing the acquisition of the stop codon in the virus genomes following passage of rBeauR-Rep-M41-Struct-2-P_3_ (P_3_) through to rBeauR-Rep-M41-Struct-2-P_25_ (P_25_). The line highlights the second nucleotide of the pair, showing that at passage 5 the rBeauR-Rep-M41-Struct-2 was a mixed population and that it appeared both substitutions occurred simultaneously and that by P_15_ rBeauR-Rep-M41-Struct-2 preferentially contained the substituted nucleotides.

In order to determine whether any nucleotide changes may have been responsible for the observed phenotypic changes of the P_25_ rIBV we decided to initially sequence the 5′-and 3′-UTRs of rBeauR-Rep-M41-Struct-2-P_25_ for comparison to the corresponding sequences of rBeauR-Rep-M41-Struct-2-P_3_ and the two parental viruses Beau-R and M41-CK. No nucleotide changes were found within the 5′- and 3′-UTR sequences of the rBeauR-Rep-M41-Struct-2-P_25_ virus indicating that the change in growth pattern was not due to one of the two UTRs changing to the other parental sequence. As a result of this finding we sequenced the complete rIBV rBeauR-Rep-M41-Struct-2-P_25_ genomic sequence and identified only three other nucleotide changes (Table 1) in addition to those identified within rBeauR-Rep-M41-Struct-2-P_3_. These new nucleotide differences corresponded to a single amino acid change in the S gene and two nucleotide changes within ORF 5b of gene 5 when comparing the M41-CK-derived sequences within rBeauR-Rep-M41-Struct-2-P_3_ and rIBV rBeauR-Rep-M41-Struct-2-P_25_ sequences; indicating that these changes arose on further passage of rBeauR-Rep-M41-Struct-2-P_3_. The two nucleotide changes within the ORF 5b sequence, nucleotides 25815 and 25816 (UU→AA), resulted in the introduction of a premature stop codon in ORF 5b and a modification of the N gene transcription regulatory sequence (TRS) from UUCUUAACAA to AACUUAACAA, the latter sequence being the more predominant IBV TRS. Sequence analysis of the rBeauR-Rep-M41-Struct-2 viruses between passages P_3_ and P_25_ (Fig. 7) indicated that at P_5_, the AA mutation was more prominent than the parental (UU) sequence and that in subsequent viruses, derived from passages P_10_, P_15_, P_20_ and P_25_, the original parental sequence gradually decreased and disappeared from detection.

## Discussion

Our previous work on the swapping of the S gene from the avirulent Beaudette (Beau-R) strain of IBV with an S gene derived from the pathogenic M41-CK isolate of IBV showed that although the S gene was responsible for tropism it was not responsible for virulence [24]–[26]. Although our results showed swapping of the S gene did not result in virulence we could not rule out the fact that other IBV structural (E, M and N) and accessory (3a, 3b, 5a and 5b) genes may also play a role and therefore be required for the acquisition of virulence. We therefore decided to exchange the region of the Beau-R genome from the end of the replicase gene to the poly(A) tail with the corresponding sequence from M41-CK to investigate whether exchange of the structural and accessory genes was sufficient to confer pathogenicity to a resultant chimaeric rIBV, rBeauR-Rep-M41-Struct. Such an approach would also allow us to determine whether or not the replicase gene plays a role in the pathogenicity of IBV. IBV Beaudette is a well known apathogenic lab strain of IBV that was attenuated by multiple, several hundred, passages in 11-day-old embryonic chicks [30], therefore loss of virulence could have resulted in multiple changes throughout the genome. IBV Beau-R is a molecular clone of Beaudette-CK [35]) that is also avirulent in chickens [26].

The objective of this study was to determine whether replacing the Beau-R structural and accessory genes with those from virulent M41-CK would result in a rIBV that was virulent when compared to Beau-R. We have used our IBV reverse genetics system [24], [25], [35] to produce chimaeric IBV, rBeauR-Rep-M41-Struct, consisting of the replicase gene from Beaudette (Beau-R) and the S, 3a, 3b, E, M, 5a, 5b, N and the 3′-UTR from M41-CK. The M41-CK-derived structural and accessory gene sequence was fused to the Beau-R replicase by homologous recombination using the TDS method [25], [37] and rIBVs were rescued in CKCs. The M41-CK-derived region, in addition to the structural and accessory genes in common with Beau-R, also contains an untranslated region, the intergenic untranslated region (IGR), between the M and gene 5, which is 305 nt in Beaudette and 350 nt in M41-CK. The M41-CK IGR, like some other strains of IBV, contains a potential open reading frame (ORF) of 285 nt potentially encoding a 94 amino acid product of 11 kD with the initiation codon immediately downstream of the M gene stop codon. A similar ORF has been identified at a similar position in the genome of turkey coronavirus (TCoV) [5], [6], the only TRS identified for TCoV is 288 nt upstream of the potential ORF, within the M gene, but with low identity to the TCoV canonical TRS. No sg mRNA for this potential ORF has been identified in TCoV or IBV, including M41-CK, infected cells. The lack of a sg mRNA, the long distance between the initiation codon and a potential TRS and the loss of the potential ORF, as a result of several deletions, in some strains of IBV indicates that the ORF is probably a pseudogene.

Two rIBVs, rBeauR-Rep-M41-Struct-2 and rBeauR-Rep-M41-Struct-12, were rescued from the rVVs, rVV-BeauR-Rep-M41-Struct-2 and rVV-BeauR-Rep-M41-Struct-12, respectively, and analysed for the presence of the M41-CK-derived sequence. Analysis of rBeauR-Rep-M41-Struct-12 identified an extra adenosine nucleotide at position 25317 in a six base polyadenosine repeat sequence within the potential 11.5 kD ORF in the M41-CK IGR, which had the potential for inactivating this potential gene product. To rule out the possibility that the loss of this potential gene product could affect any pathogenicity of this virus we decided to proceed with rBeauR-Rep-M41-Struct-2, which had the correct sequence, for subsequent experiments.

We have characterised rBeauR-Rep-M41-Struct-2-P_3_ *in vitro*, *ex vivo* and tested its pathogenicity *in vivo* using one-day-old- chickens. The overall growth pattern of rBeauR-Rep-M41-Struct-2 in CKCs was more similar to that of Beau-R (Fig. 2) except it grew to a titre of about 1 log_10_ less throughout the growth cycle. The main objective of this study was to determine whether swapping of the structural and accessory genes would restore virulence. To this effect we used rBeauR-Rep-M41-Struct-2 to infect one-day-old chicks, and used clinical signs and measurement of the ciliary activity of the epithelial cells lining the trachea of the infected chickens to assess any pathogenicity associated with the virus. No clinical signs, snicking, wheezing and nasal discharge, associated with an IBV infection were observed in the chickens infected with either rBeauR-Rep-M41-Struct-2 or Beau-R; the latter as in previous experiments did not show any clinical signs [26]. In contrast, chickens infected with M41-CK did show clinical signs of an IBV infection (Fig. 3); as shown previously [26]. These observations demonstrated that rBeauR-Rep-M41-Struct-2 was not pathogenic indicating that replacement of the structural and accessory genes did not restore virulence.

Analysis of epithelial cells, removed from the tracheas of the infected chickens, for the presence of infectious IBV using TOCs failed to detect virus from chickens infected with either Beau-R or rBeauR-Rep-M41-Struct-2. In contrast, epithelial cells from chickens infected with M41-CK showed the presence of virus with a titre of 4 log_10_ CD_50_ in TOCs, demonstrating that M41-CK was present in the tracheas of the infected chickens. Analysis of the epithelial cells for the presence of any IBV-derived RNA by RT-PCR also indicated that no Beau-R or rBeauR-Rep-M41-Struct-2 was present or was below detection in the epithelial cells examined; this was in contrast to the cells examined from chickens infected with M41-CK, in which virus-derived RNA was detected by RT-PCR (Fig. 4).

Subsequent analysis of rBeauR-Rep-M41-Struct-2 *ex vivo* using TOCs surprisingly showed that the rIBV did not cause ciliostasis when directly used to infect TOCs, in contrast to Beau-R and M41-CK which caused ciliostasis. This observation raised the possibility that the lack of ciliostasis and detection of rBeauR-Rep-M41-Struct-2 in the tracheal epithelial cells of chickens infected by the virus using TOCs for our pathogenicity experiments may have been interpreted incorrectly as the read out for these tests was ciliostasis. However, it should be noted no clinical signs were observed nor was any rBeauR-Rep-M41-Struct-2-derived RNA detected. Growth analysis of rBeauR-Rep-M41-Struct-2 in TOCs showed that the lack of ciliostasis observed with rBeauR-Rep-M41-Struct-2 was not due to the inability of the virus to replicate in TOCs (Fig. 5). The rIBV rBeauR-Rep-M41-Struct-2 was able to replicate in TOCs, though the amount of virus produced was about 1 log_10_ less than for the growth of Beau-R and M41-CK, somewhat analogous to the growth pattern observed for the virus on CKCs.

IBV rBeauR-Rep-M41-Struct-2 consisted of the Beau-R replicase and M41 structural and accessory genes and as a consequence had a 5′-UTR derived from Beau-R and a 3′-UTR from M41. There are known differences between IBV 3′ UTRs, in fact the M41-CK 3′-UTR is 184 nt smaller than the Beau-R 3′-UTR, the remaining part of the M41-CK 3′-UTR representing the conserved region of the IBV 3′-UTR and contains the predicted RNA secondary structures believed to be involved in replication [44]. There are 7 and 3 nucleotide differences between the 5′- and the conserved region of the IBV 3′-UTRs, respectively, of Beau-R and M41-CK ([33], [42], raising the possibility that if the two UTRs interact during replication their heterologous nature could be responsible for the observed decrease in growth for rBeauR-Rep-M41-Struct-2 in both CKCs and TOCs. Impairment in growth on TOCs may also have been responsible for the loss of ciliostasis. We hypothesised that an increase in growth would be a selective advantage to the virus and therefore decided to serially passage rBeauR-Rep-M41-Struct-2 25 times on CKCs to see whether any adaption could result in a higher growth rate and a virus that could cause ciliostasis in TOCs. For example, due to the very few differences in the 5′- and 3′-UTRs, between the two parental viruses, a nucleotide substitution in either UTR that may result in a more homogenous interaction, as seen with either parental virus, may result in an increase growth rate. Analysis of the passaged virus, rBeauR-Rep-M41-Struct-2-P_25_, on CKCs and TOCs showed an altered growth rate in comparison to rBeauR-Rep-M41-Struct-2-P_3_ (Fig. 6). However, rBeauR-Rep-M41-Struct-2-P_25_ still did not cause ciliostasis in TOCs. Sequence analysis of the 5′- and 3′-UTRs of rBeauR-Rep-M41-Struct-2-P_25_ showed that there were no nucleotide substitutions within these regions when compared to rBeauR-Rep-M41-Struct-2-P_3_, indicating that the change in the growth characteristics was not associated with changes in the UTRs. We therefore sequenced the entire genome of rBeauR-Rep-M41-Struct-2-P_25_ for comparison with the sequence in rVV-BeauR-Rep-M41-Struct, used for generating rBeauR-Rep-M41-Struct-2-P_3_, in order to identify any potential changes that may have been responsible for the phenotypic change in growth. We identified three nucleotide changes between the P_3_ and P_25_ viruses, one resulted in an amino acid change, Phe→Leu, in the S1 region of the S gene and two adjacent nucleotides in the ORF-5b sequence that had two potential effects, (1) the introduction of a premature stop codon in ORF-5b and (2) modification the N gene TRS. These observations indicate that the increase in growth associated with rBeauR-Rep-M41-Struct-2-P_25_ could have arisen from the amino acid change in the S protein, the loss of ORF-5b or a potential change in the expression levels of sg mRNA 6, which is responsible for the expression of the N protein. Overall, the main conclusion from our results is the fact that rBeauR-Rep-M41-Struct-2 was non-pathogenic; indicating that the loss of virulence associated with Beaudette is not determined by the structural and the accessory genes of IBV but resides within the replicase gene.

Our results demonstrating that the IBV replicase gene is a determinant of pathogenicity differs from the conclusion by Navas-Martin *et al*. [45] who reported that the differences in pathogenicity between two strains of the murine coronavirus mouse hepatitis virus (MHV), MHV-JHM and MHV-A59, mapped to the MHV genome encoding the structural and accessory genes downstream of the haemagglutinin esterase (HE) gene. The replicase gene was interchangeable between the two virus genomes but the differences in pathogenicities associated with two viruses did not appear to correlate with the replicase genes of the rMHVs generated. However, a major difference between the two MHV isolates and the two IBV viruses we used was that the two MHVs were pathogenic but demonstrated different pathogenicities, neurovirulence and hepatitis, respectively, whereas the difference between the two IBV isolates used in this work was that one causes disease and the other does not. It is clear from the MHV work that both replicase sequences are of a “virulent” phenotype and that the pathogenicity phenotype of the recombinant viruses is associated with the structural and accessory genes. In contrast our results demonstrated that loss of virulence *per se* can be determined by some attenuating modification to one or more of the coronavirus replicase components. Recent work involving the attenuation of a nephropathogenic strain of IBV by serial passage in embryonated chicken eggs, reported that the virus became fully attenuated by passage P_110_ [46]. Sequence analysis of the 3′-7 kb (S gene to poly(A) tail) region of the genome from the P_110_ virus identified several amino acid substitutions and a 109 nt deletion within the 3′ UTR when compared to P_0_ virus. The authors reported that the changes identified were potentially responsible for attenuation; they did not report any changes within the replicase gene of the P_110_ virus and indicated that other changes, apart from those identified in the 3′-7 kb region, within the genome may also be involved in attenuation. Other workers attenuated the virulent Ark DPI 11 strain of IBV following 101 passages in embryonated eggs (Ark DPI 101) and compared the complete genomes of both viruses; identifying 21 nucleotide changes corresponding to 17 amino acid changes [47]. The nucleotide changes resulted in eight amino acid changes in Nsp2 (1), Nsp3 (3), Nsp6 (2), Nsp10 (1) and Nsp13 (1) of the replicase protein, eight amino acid changes in the S protein and one amino acid in ORF 5a and the N protein. The authors were unable to confirm which amino acid changes were responsible for loss of pathogenicity. Taking into consideration our results that loss of virulence associated with the Beaudette strain resides in the replicase and the results of Ammayappan *et al.* [47], it is possible that very few amino acid substitutions within the replicase gene can result in attenuation following serial passage in embryonated eggs.

The replicase gene of IBV encodes 15 Nsps, some of them with known enzymatic functions [48]. How these proteins function in the context of pathogenesis is still not well understood, however, some of the Nsps for other coronaviruses have been linked to loss of virulence. For example, the loss of Nsp1 from MHV did not affect replication in tissue culture but severely attenuated the rMHV *in vivo* [49]; inactivation of the MHV ADP-ribose-1″phosphatase activity in Nsp3 caused a reduction in virus replication in the livers of infected mice but did not induce liver disease [50] and a single amino acid change in the MHV Nsp14 did not alter the replication of the rMHV in tissue culture but resulted in attenuation in mice [51].

Our previous spike swapping results demonstrated that introduction of an S protein from a virulent isolate of IBV did not confer virulence on Beau-R indicating that loss of virulence was not receptor-mediated. Results from work described here has shown that replacing all the Beaudette structural and accessory proteins with those from a virulent isolate of IBV did not restore virulence. Generation of the rIBV produced in this work can either be viewed as replacing the structural and accessory genes of an avirulent virus with those of a virulent virus or replacing the replicase gene of a virulent isolate (M41-CK) with one from an avirulent virus (Beau-R). In either scenario our results indicate that loss of virulence associated with IBV Beaudette resides within one or more of the 15 IBV replicase proteins comprising the IBV replicase gene.
